# Comparison of covariate selection methods with correlated covariates: prior information versus data information, or a mixture of both?

**DOI:** 10.1007/s10928-020-09700-5

**Published:** 2020-07-13

**Authors:** Estelle Chasseloup, Gunnar Yngman, Mats O. Karlsson

**Affiliations:** Department of Pharmaceutical Biosciences, Faculty of Pharmacy, Uppsala University, Room BMC B3:4 Biomedicinskt centrum BMC, Husargatan 3, Box 591, 751 24 Uppsala, Sweden

**Keywords:** Covariates, Prior, Correlation, Stepwise covariate modelling, Full fixed effects modelling, Prior-adjusted covariate selection

## Abstract

The inclusion of covariates in population models during drug development is a key step to understanding drug variability and support dosage regimen proposal, but high correlation among covariates often complicates the identification of the true covariate. We compared three covariate selection methods balancing data information and prior knowledge: (1) full fixed effect modelling (FFEM), with covariate selection prior to data analysis, (2) simplified stepwise covariate modelling (sSCM), data driven selection only, and (3) Prior-Adjusted Covariate Selection (PACS) mixing both. PACS penalizes the a priori less likely covariate model by adding to its objective function value (OFV) a prior probability-derived constant: $2*{\kern 1pt} \,{\ln}\left( {{\Pr}\left( X \right)/\left( {1 - {\Pr}\left( X \right)} \right)} \right)$, Pr(X) being the probability of the more likely covariate. Simulations were performed to compare their external performance (average OFV in a validation dataset of 10,000 subjects) in selecting the true covariate between two highly correlated covariates: 0.5, 0.7, or 0.9, after a training step on datasets of 12, 25 or 100 subjects (increasing power). With low power data no method was superior, except FFEM when associated with highly correlated covariates ($r=0.9$), sSCM and PACS suffering both from selection bias. For high power data, PACS and sSCM performed similarly, both superior to FFEM. PACS is an alternative for covariate selection considering both the expected power to identify an anticipated covariate relation and the probability of prior information being correct. A proposed strategy is to use FFEM whenever the expected power to distinguish between contending models is < 80%, PACS when > 80% but < 100%, and SCM when the expected power is 100%.

**Electronic supplementary material:**

The online version of this article (10.1007/s10928-020-09700-5) contains supplementary material, which is available to authorized users.

## Introduction

Population models combine pharmacological and statistical models to describe biological processes. They allow the distinction between the typical values (fixed effects) and the variability (random effects) of the model parameters. The inclusion of explanatory covariates in such models can decrease the unexplained variability of a parameter, increase the precision of the estimates, and add a mechanistic interpretation. Covariate inclusion is often supported by a physiological rational (e.g. the enzyme genotype in metabolism). Building a covariate model can confirm some prior knowledge, provide new information, support dose adjustment, and help in the design of upcoming experiments.

Numerous approaches have been developed in order to identify covariates for population models [1, 2], for example, visual inspection of unexplained parameters variability versus covariates plots [3], generalized additive models [4], Stepwise Covariate Modelling (SCM) [5], Wald’s approximation method [6], Full Fixed Effects Modelling (FFEM) [7, 8] or Full Random Effects Modelling [9–11]. The different methods place different emphasis on prior knowledge versus information in the present data when selecting covariates.

Correlated covariates are common in biology (e.g. body weight and body surface area, or age and renal function). Prior knowledge is often not sufficient to identify with certainty which of the correlated covariates is the more closely related to the parameter in question. The selection of the less predictive covariate can impact both the model performance (parameter precision and stability issues [12]) and the interpretation of the results which could be misleading during the learning process of drug development. The selection between correlated covariates based on data alone may be informative when data are rich in information on the parameter and covariate (high power case), but when information is poor (low power case) selection bias may result in poor predictive performance of the developed model.

In this work, we will compare three covariates selection methods according different importance to the main selection rationales that are prior information or data information. (1) FFEM requires a covariate pre-selection and do not account for data information, (2) sSCM is a simplified version of the data driven SCM method which do not use any prior information, and (3) Prior Adjusted Covariate Selection (PACS) a new selection method balancing pre-selection (or prior information) and the data information. The aim is to illustrate their relative merits when applied to external data. For this purpose, a simple situation was investigated with only one parameter and two correlated covariates.

## Material and methods

In order to compare the three covariates selection methods, data were simulated from a simple model according to the workflow presented below.

### Software packages

The simulations were performed with PsN version 4.8.8 [13], driving NONMEM version 7.4.3 [14]. The covariate selection methods were implemented in the statistical software R, version 3.6.0 [15].

### Models

Two covariates, A and B, were simulated for every simulated subject. These were variables normally distributed with a mean of zero, a variance of 0.045, and a correlation varying across different scenarios (0.5, 0.7, or 0.9). The model used to simulate the data is a simple constant-rate infusion (rate = 1) steady-state model involving clearance as the only pharmacokinetic parameter ($CL$), and including one of the covariates ($COV=A$ or $B$) depending on which was the true covariate (Eq. 1):

1 $$\begin{array}{*{20}r} \hfill {{\log}\left( {CL} \right) = {\log}\left( {\theta_{1} } \right) + \eta_{1} + COV} \\ \hfill {Y = - {\log}\left( {{\exp}\left( {CL} \right)} \right) + \varepsilon } \\ \end{array}$$

where ${\theta }_{1}$ is the typical value of the clearance and Y the observed data. The unexplained interindividual variability (IIV) variance of the random effect, ${\eta }_{1}$, was set to 0.045, making the total parameter variability circa 30%, with the covariate (A or B) explaining half of the total parameter variability. No covariance between ${\eta }_{1}$ and COV was included in the simulations. The residual random variability was also set to 30% ($\varepsilon \sim \mathcal{N}(0,0.09)$).

Three covariate models were estimated based on the simulated data. The model ${M}_{0}$ with no covariates (Eq. 2; only used for assessing power, see below), the models ${M}_{A}$ and ${M}_{B}$ with only the covariate $A$ (Eq. 3) or only the covariate $B$ (Eq. 4).

2 $${\log}\left( {CL} \right) = {\log}\left( {\theta_{1} } \right) + \eta_{1}$$

3 $${\log}\left( {CL} \right) = {\log}\left( {\theta_{1} } \right) + \eta_{1} + COV_{A} \cdot \theta_{A}$$

4 $${\log}\left( {CL} \right) = {\log}\left( {\theta_{1} } \right) + \eta_{1} + COV_{B} \cdot \theta_{B}$$

where ${\theta }_{A}$ and ${\theta }_{B}$ are the covariate effects. The selection process was made between the models ${M}_{A}$ and ${M}_{B}$ only, to always arrive at a model with exactly one covariate explaining variability in CL. The simultaneous inclusion of two correlated covariates on the same parameter is usually avoided in covariate model building and none of the scenarios here allowed such models.

### Covariates selection methods

#### Prior information selection: FFEM

In the FFEM selection method, the covariates are pre-selected by the modeler and included in the model as fixed effects. It is a data-agnostic method where the selection process is independent of the information in the modelled data: the covariate model is defined a priori.

The performance of the FFEM method is expected to be related to the probability with which the true covariate was a priori selected. To investigate the quality of pre-selection of the modeler, we used 11 different values (0, 0.1, …, 1) for the probability of the selected covariate $X$, to be the true covariate $P(X)$.

#### Data driven selection: sSCM

SCM is a data driven selection method since the selection process is solely based on goodness-of-fit (i.e. OFV): the covariates are included into the model when the decrease in OFV is significant according to a chi-square distribution for a given p-value (likelihood ratio test). The covariate selection is done step by step, adding only one covariate per step, starting with the model without any covariate (base model). At each step, all the covariates not already included are tested, and among the significant ones, the one decreasing the most the OFV is selected for the next step. Usually, a backward step with a lower p-value is performed at the end of the inclusion process, removing the covariates one by one to check for any over parameterization.

In this case, we wanted to compare model selection methods when the model scope was the same. For this purpose, the method was restricted to compare the two possible models (sSCM): the selection was thus made between ${M}_{A}$ and ${M}_{B}$, by simply selecting the model with the lowest OFV, which is equivalent to selection according to the Akaike Information Criteria (AIC).

#### Prior information and data-based selection: PACS

This selection method accounts both for the data information (OFV), and the prior information. The prior information is introduced as penalty added to the OFV value of the model that appears less likely to the modeler. The value of this penalty is set according to the following formula:

5 $$2\,{*}\,{\ln}\left( {\frac{{{\Pr}\left( X \right)}}{{1 - {\Pr}\left( X \right)}}} \right)$$

where $Pr(X)$ is the probability for the covariate X to be the best of the two covariates (A or B), as assigned by the modeler. In the simulations, we only explore $0.5\le Pr(X)\le 0.999$, since $Pr(X)<0.5$ is equivalent when interchanging A and B. In this context PACS is equivalent to SCM when $Pr(X)=0.5$ (i.e. the penalty is null), and PACS is equivalent to FFEM when $Pr(X)=1$ (i.e. the penalty is infinite for the model without the pre-selected covariate). After the addition of the penalty, model selection occurs in the same manner as for the sSCM.

### Workflow

**Fig. 1:**
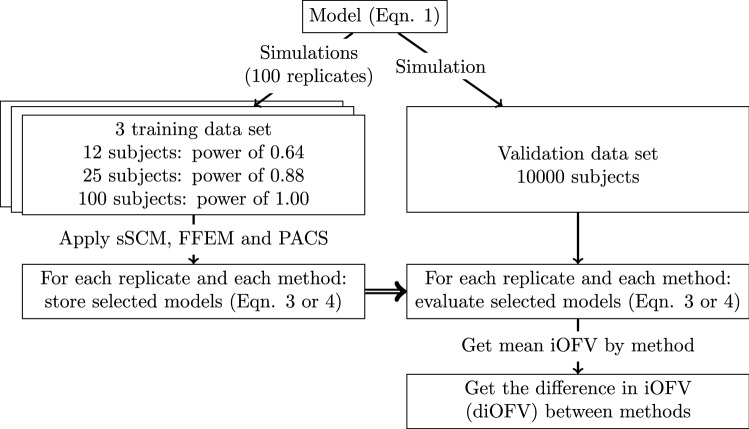
Workflow used to simulate the data and analyze the results. *iOFV *individual objective function value, *diOFV *difference in individual objective function value

To assess the predictive performances of the three covariates selection methods, the data were generated according to the workflow presented in Fig. 1. Data were simulated (100 replicates) for three training datasets of 12, 25, and 100 individuals (two observations per subject), corresponding respectively to a power of $0.64$, $0.88$, and $1.00$. The power was calculated here as the frequency with which the true covariate model was significantly better than the model without covariate (${M}_{0}$) using the likelihood ratio test with one degree of freedom. A validation dataset of 10,000 individuals (two observations per subject) was also simulated for each scenario.

FFEM, sSCM, and PACS were applied to each replicate of each training dataset. The selected models were retained to evaluate them on the corresponding validation dataset. In this evaluation, the individual objective function value (iOFV) was obtained using, without reestimation, the parameters from the model developed based on the training data. The difference in the mean iOFV between methods was then used to compare the external evaluation performances of the three methods. The mean iOFV was used here as it may offer a slightly more easily interpreted value compared to the difference in total OFV for a very large data set.

## Results

**Fig. 2:**
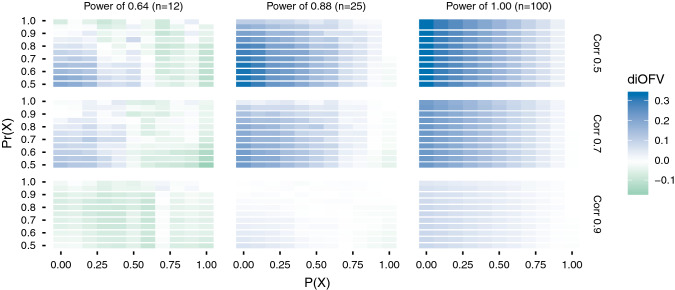
Difference in iOFV (diOFV) in the validation data set between FFEM and PACS for different power and covariate correlation combinations simulated. The y-axis is the prior used to favor the covariate X (in PACS); the x-axis is the probability of X to be the true covariate. Green (blue) color indicates that FFEM (PACS) is superior (Color figure online)

**Fig. 3:**
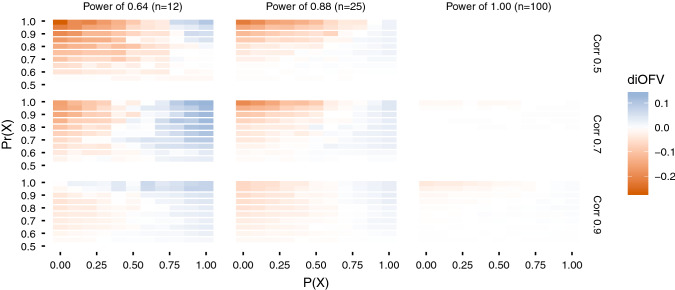
Difference in iOFV (diOFV) in the validation data set between sSCM and PACS for different power and covariate correlation combinations simulated. The y-axis is the prior used to favor the covariate X (in PACS); the x-axis is the probability of X to be the true covariate. Red (blue) color indicates that sSCM (PACS) is superior (Color figure online)

**Fig. 4:**
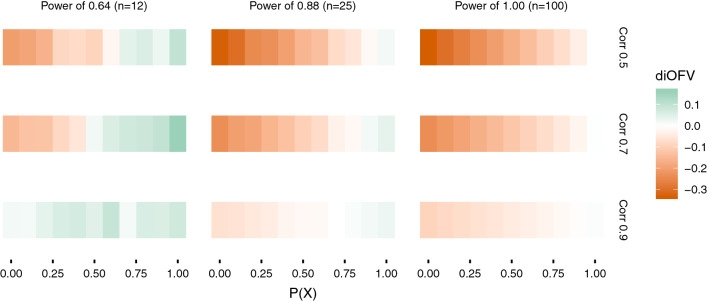
Difference in iOFV (diOFV) in the validation data set between sSCM and FFEM for different power and covariate correlation combinations simulated. The x-axis is the probability of X to be the true covariate. Red (green) color indicates that sSCM (FFEM) is superior (Color figure online)

**Fig. 5:**
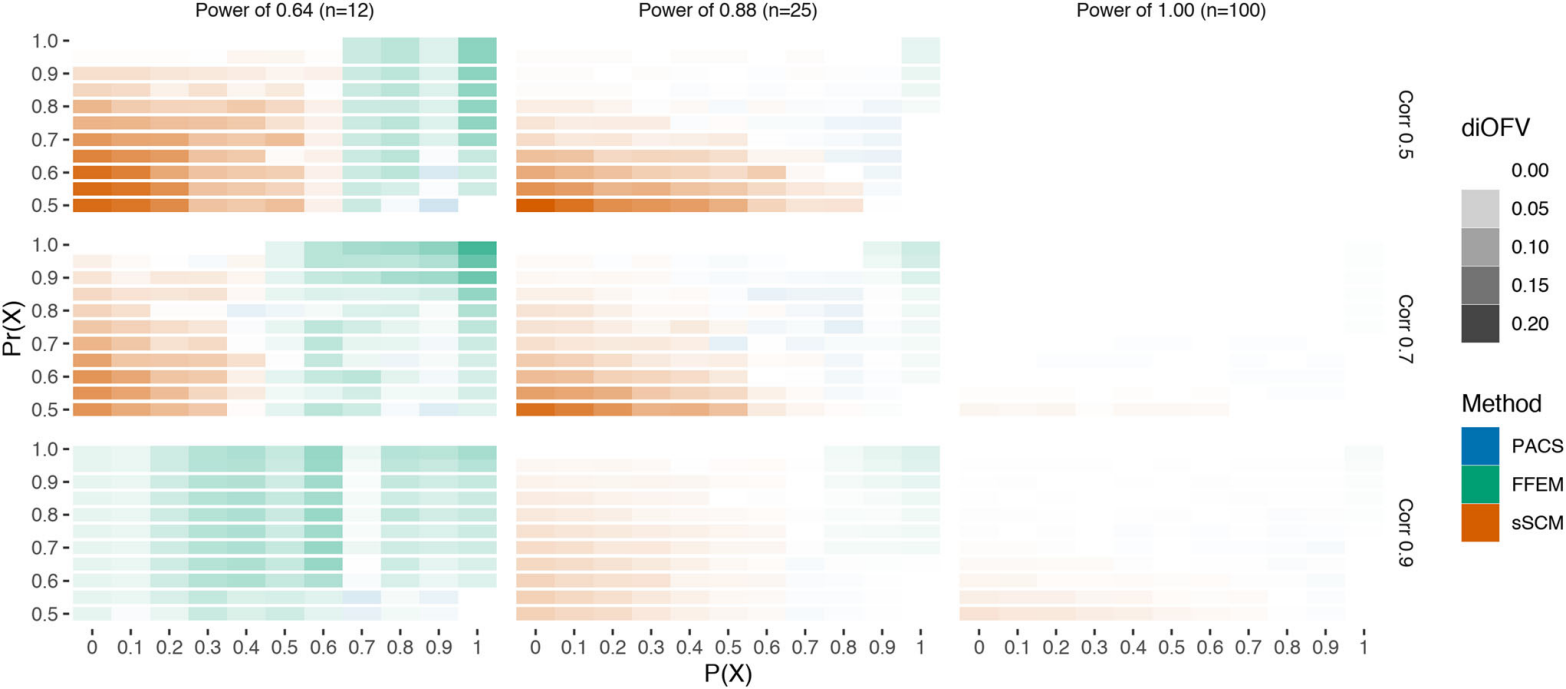
Difference in iOFV (diOFV) between the methods providing the model with the lowest and second-lowest iOFV in the validation data set for different power and covariate correlation combinations simulated. The x-axis is the probability of X to be the true covariate

**Fig. 6:**
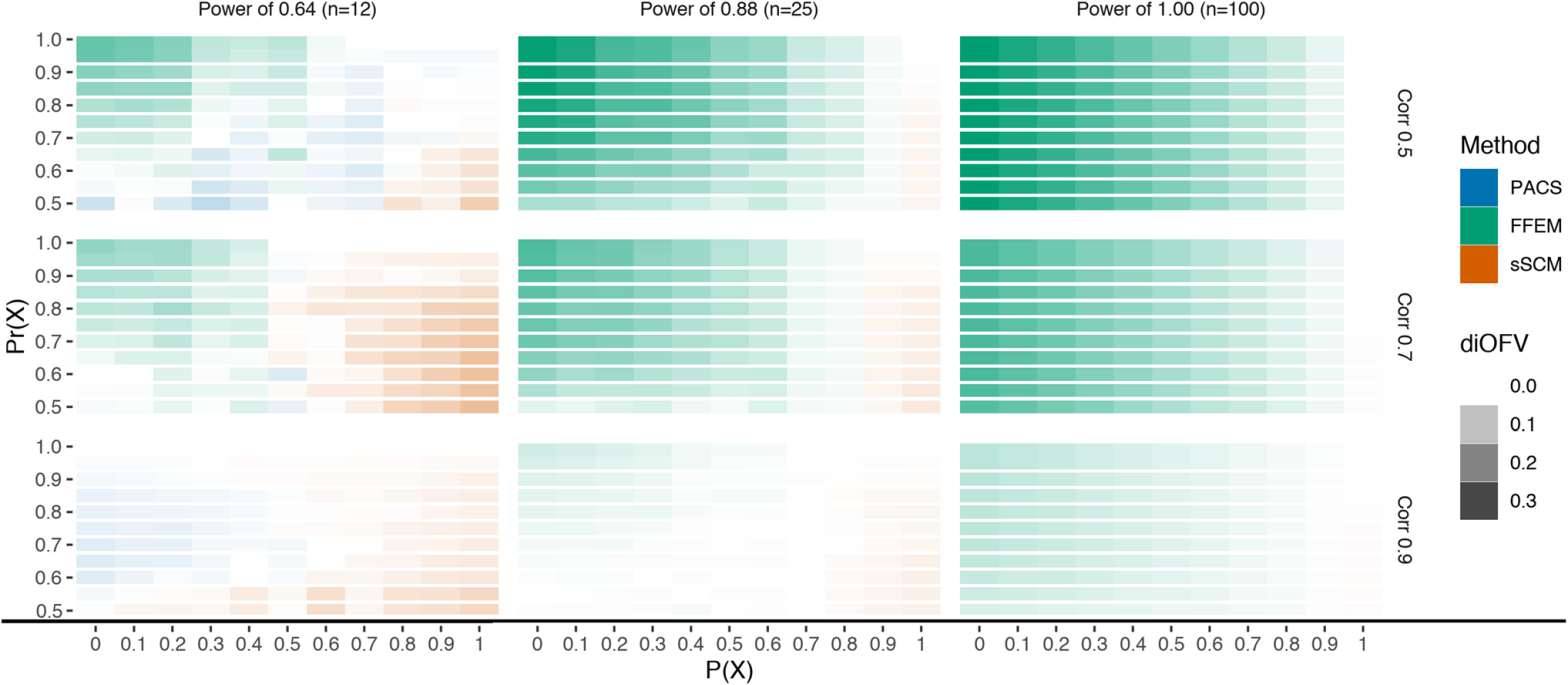
Difference in iOFV (diOFV) between the methods providing the model with the highest and second-highest iOFV in the validation data set for different power and covariate correlation combinations simulated. The x-axis is the probability of X to be the true covariate

The results are presented as heat map plots where green, red, and blue colors indicate superior performance of FFEM, sSCM, and PACS, respectively. The color gradient reflects the differences in the average iOFV (diOFV) between each pair of covariate selection methods for Figs. 2, 3, and 4. The gradient represents the diOFV between the methods providing the best and second-best models to describe the validation data set in Fig. 5, and the diOFV between the methods providing the least and second least good models to describe the validation data set in Fig. 6. Hence the Figs. 5 and 6 compare the three methods simultaneously.

The comparison between PACS and FFEM (Fig. 2) showed an overall superiority of PACS, except when the power was low and the correlation close to 1. For the three powers ($0.64$, $0.88$, and $1.00$), the differences decreased as the correlation between the covariates increased. A prior-adjustment was less valuable when the modeler was more likely to make the right choice (i.e. $P\left(X\right)$ is high). For the high power, the better performances of PACS can be explained both by the additional information brought by the prior, and the data information. When the data were not informative (lowest power) and covariates were highly correlated, the FFEM performed better across most values for P(X) and Pr(X).

PACS and sSCM (Fig. 3) showed very similar performance when the data were highly informative (power of 1.0). For the other scenarios, PACS in general performed better when P(X) was above 0.5, that is the true covariate was favored in PACS, whereas the opposite was true for P(X) below 0.5. In the low power case, smaller differences were observed when the covariates were highly correlated.

The comparison between FFEM and sSCM is illustrated in Fig. 4. As in Figs. 2 and 3, the differences between the two methods decreased as the correlation increased. sSCM performed better than FFEM with informative data (high power). In the lowest power case, FFEM had better performances when the pre-selected covariate was the true one. With high correlation and low power, even when favoring the wrong covariate ($P\left(X\right)<0.5$), FFEM performed better than sSCM.

Figure 5 showed the methods providing the covariate model in best agreement with the validation data set and its closeness to the second-best method. With rich data (power of 1), the two best methods (PACS and sSCM) were equivalent, their difference with the method providing the least descriptive model (FFEM) increased when the probability to select the true covariate P(X) decreased (Fig. 6). However, FFEM performed better when P(X) = 1, hence when the right covariate was always selected with FFEM. When the data are not informative (power of 0.64), and with highly correlated covariates (correlation of 0.9), FFEM was the best method. With lower covariate correlations however, sSCM provided the best model for low values of P(X). Figure 6 showed that PACS is very seldom providing the worst model for the validation data, contrary to FFEM, particularly predominant when the data are informative (high power).

## Discussion

The aim of this study was to compare the relative performance of the covariate selection methods in the case of correlated covariates. FFEM and sSCM were selected for inclusion in the comparison as they are standard methods and have a clear rationale for selection, prior information, and data information, respectively. PACS is balancing between these two extremes by allowing the OFV used for selection to be influenced by both the data and the prior. This simple simulated example with one parameter and two correlated covariates was designed to illustrate the main properties of these selection methods without potential confounding factors.

The comparison was done across different scenarios with respect to (i) informativeness of data (low to high power), (ii) probability that the true covariate was identified a priori (P(X)), (iii) correlation between the two covariates (moderate to high) and, for PACS only, (iv) the probability for the modeler to favor the correct. The relative performance of different methods was strongly related to these aspects as discussed below.

The most important aspect of the relative performance of the three methods was the power. In the high power situation, sSCM and PACS performed best and FFEM performed worst unless the probability of selecting the correct covariate was one, in which case all three methods performed essentially the same. This conclusion is similar to the one proposed by Khandelwal et al. [16]. With moderate power, a similar pattern was found, although sSCM showed superiority over PACS when the modeler was incorrect in the pre-selection of the covariate. For the low power situation, relations are more complex and depend on the correlation between the two covariates. With high correlation between the covariates and low power, FFEM is the best method regardless of whether the pre-selection was correct or incorrect. This may appear as counterintuitive, but can be understood from the selection bias associated with sSCM and PACS. The selection process will favor the covariate showing the stronger relation with the parameter and when the two covariates are highly correlated, this will typically result in a covariate coefficient that is biased to have a too high absolute value. With low power and lower (0.7 or 0.5) correlation between the covariates, FFEM is the best method when the pre-selected covariate is the better one. When this is not the case sSCM is the best method.

The above relations offer support to some common covariate modelling strategies. For example to preselect one of two highly correlated covariates, such as body weight and body surface area, when the number of studied subjects is limited [17]. Another example is the attempted identification of the superior of two correlated covariates when the data are highly informative [16]. However, the results point to a strategy that may be favorable for covariate model building in general. In such a strategy, the method for covariate modelling, FFEM, sSCM, or PACS, would be preselected based on the power to discriminate between models and any prior knowledge regarding covariate relations. Factors affecting the power to discriminate between models include study design, covariate distributions, and expected strength of the covariate relations. The study design (e.g. number of subjects and number and timing of observations) will impact the information about the parameter(s) in question. The expected covariate variability and expected covariate-parameter strength will further inform on the expected power to identify a covariate relation, and the correlation between the covariates will provide a basis for assessing selection bias. Thus, for a given situation, the relative merits of different covariate modelling strategies can be assessed before covariate-parameter relationships are explored and tailored to the situation in question. To our knowledge, such a strategy has not been implemented in real data analysis examples and would come with extra time and cost of analysis compared to traditional covariate modelling. However, enhanced discrimination between candidate covariates and/or predictive precision may make such investments justified. In short, such a strategy could be summarized as: (i) if the power to discriminate between the covariates is complete (i.e. 100%) perform data driven selection, (ii) if the power is low (< 80%) use FFEM, and (iii) if the power is 80–100%, use PACS if one covariate is deemed more likely than the other, otherwise use purely data driven selection. An illustration of this strategy using a real data example is available in the Online Appendix 1. The comparison of this strategy with the three other covariate selection methods, using the simulated example used in this paper, resulted in mean iOFV across all scenarios of − 17.92 (FFEM), − 18.06 (sSCM), − 17.99 (PACS), and − 18.19 (power-based strategy). PACS, when the correct covariate was given any prior probability above 0.5, had a corresponding value of − 18.23. Additionally, the best (true) model and the worst (false) models had values of − 18.48 and − 16.21, respectively.

The example used to illustrate and compare the methods was very simple. One aspect is that the model only contains one parameter. With multiple parameters in a model, the selection complexity is increased as there is a question with which parameter(s) a covariate may correlate with. The inclusion, or not, of a covariate can influence the power and estimated strength of the same covariate on another parameter [11]. For example, if a covariate influences two parameters, but is included only on one, this relationship is expected to be biased [11]. Another simplification is that in the data driven modelling only two contending covariate models were compared. This mimics a situation where there is a known relation with an underlying feature, e.g. body size, but lack of certainty on how this is best represented, e.g. by total or lean body weight. The addition of the possibility to include neither covariate in the model, which is more common for data driven covariate modelling, would mainly impact the result of the low power situations studied. Neither did we allow both covariates to enter the model simultaneously. This mimics common present practice. In the present simulations, the power to identify and discriminate between the covariates was varied by varying the number of subjects but other simulations settings could similarly impact the power. A higher power could have been achieved by increasing the explanatory value of the covariates, decreasing the unexplained variability, increasing the number of observations per subject, or decreasing the residual variability. When a covariate selection is dependent on an improvement in goodness-of-fit, as when full and reduced models are compared using the likelihood ratio test, the power is also dependent on the significance criteria. The higher the demand for improvement, for example through a low p-value, the lower the power of identifying a covariate relation. To learn in detail about the relative merits of the different methods for real data analysis problems, simulation studies tailored for the situations are recommended.

In this work, we introduced PACS. It showed some advantages relative to sSCM and FFEM, but was the overall best method only in some cases when Pr(X) was close to P(X), i.e. when the prior was close to the true probability. When power was high, sSCM performed as well, and when power was low either sSCM or FFEM performed better. Noteworthy is that PACS was the worst method less often than either sSCM or FFEM and thus may offer robustness. In this implementation, we used a prior suited for maximum likelihood estimation, a so-called “frequentist” prior [18]. PACS could also be implemented as a Bayesian prior, but such explorations were not performed as SCM procedures typically use maximum likelihood. In this implementation, the prior in PACS was used to inform the relative likelihood of two parameters being related to a parameter. In the case of a single covariate relationship, the relative probability of an existing versus a non-existing relationship could be used instead. As in the present work, a value of such a relative probability approaching one would collapse PACS towards FFEM. However, as long as the relative probability is slightly lower than one, it allows the data, when highly informative, to contribute to the covariate inclusion. This latter situation may be a favorable feature of PACS.

## Conclusion

The selection of covariate relations based on both prior information and/or data can be rationally motivated depending on the situation. The existing covariates selection methods are based only on pre-selection (e.g. FFEM), or data information (e.g. SCM). The introduction of a prior in the selection process is a new feature allowing PACS to combine the two, which provides an interesting alternative. The method(s) performing the best are mainly determined by the power, where high power favors SCM and PACS and low power FFEM. Thus the selection of a strategy for covariate model building based on the power of discrimination can be expected to perform better than one that only considers the correlation between covariates.

## Electronic supplementary material

Below is the link to the electronic supplementary material.

Supplementary file1 (PDF 241 kb)
