# Supplementary material for: Comparison of covariate selection methods with correlated covariates: prior information versus data information, or a mixture of both?
Source: J Pharmacokinet Pharmacodyn. 2020 Jul 13;47(5):485–92. doi: 10.1007/s10928-020-09700-5 (PMC7520415; doi:10.1007/s10928-020-09700-5)
Supplement: Supplementary file 1 — Supplementary file1 (PDF 241 kb) [file 10928_2020_9700_MOESM1_ESM.pdf]

Appendix 1: A case study to illustrate a decision about covariate modelling strategy

Data

Moxonidine concentrations and associated correlated covariates (r=0.69), weight (WT) and creatinine clearance (CRCL) from 74 patients were used (Karlsson et al. 1998).

	CRCL	WT
Mean	67.7	78.5
Std.Dev	21.8	15.8
Min	35.0	41.0
Max	142.0	125.0
Median	65.0	77.0
IQR	30.5	19.8

```
ggpairs(covariates, lower = list(continuous = "smooth"))+
ggtitle( "Scatter plot of the correlated covariates with correlation" )
```

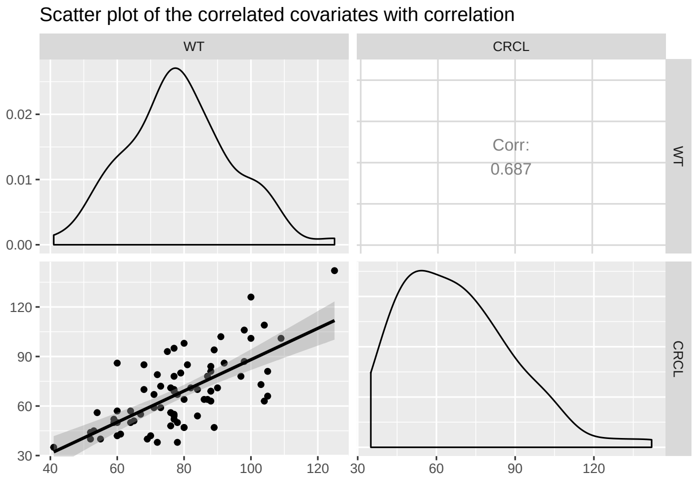

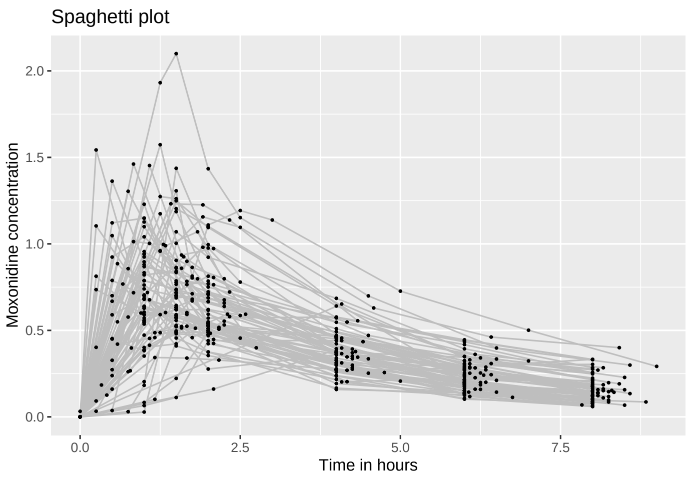

Selection of the covariate modelling strategy

For the pharmacokinetic model of the moxonidine concentration-time data in congestive heart failure patients (Karlsson et al. 1998), there are two covariates of particular interest to describe the variability of clearance: weight (WT) and creatinine clearance (CRCL). These two covariates are highly correlated (r=0.69). The description of the data is provided above in the Data section.

The decision of the covariate selection method to use (FFEM, sSCM, or PACS) depends on the expected power of the data to (1) select a covariate versus no covariate, and (2) discriminate between two correlated covariates. As discussed in the paper, FFEM would be more appropriate for a low power case, but for data with higher power sSCM or PACS would be more interesting and allow usage of the data information.

In order to select the more appropriate covariate method for the moxonidine data, power was computed via a model-based simulation approach, comparing the pharmacokinetic model without covariates M_0 and the two covariate models M_{CRCL} and M_{WT}. These covariate models were based on prior expectations and the variability in CL originating from the covariate component was similar for the two sets of simulation.

- M_{CRCL}: linear model with creatinine clearance assuming 50% of the elimination is renal for the typical patient.
- M_{WT}: power model, with the allometric exponent set to 0.75.

Stochastic simulations ($n = 1000$) were performed with each one of the two covariate models, and estimated with all three models (no covariate, CRCL and WT), including estimation of the covariate-related parameters. As shown in the table below, the power is high, both to select each covariate model over no covariate, and to select the true covariate (used in the simulation) over the correlated one. The power was computed with the LRT when the models were nested ($\alpha = 0.05$), and with the AIC when not.

Simulation model	Compared models	Power	Expected dOFV
M_{CRCL}	M_0	97.5%	25.0
M_{CRCL}	M_{WT}	92.6%	11.1
M_{WT}	M_0	97.6%	24.1
M_{WT}	M_{CRCL}	93.1%	10.5

With such power to detect the assumed covariate relationships (>80% but <100%), PACS is selected. Knowing that moxonidine is to a large extent renally cleared, creatinine clearance will be favoured over weight. PACS with a prior probability of 0.75 favouring CRCL over WT results in a modelling strategy which accounts for the fact that the data are largely expected to discriminate between the two covariates, but additionally

incorporating the prior expectation of CLCR being more likely. Numerically, a prior of 0.75 translates to an OFV penalty of $2 * ln(0.75/0.25) = 2.19$ for the WT model.

Models

Following the fit of the three models to the patient data, the PACS strategy identified the model incorporating CRCL as a covariate on moxonidine CL superior over the alternative models. The difference in OFV between the no covariate and covariate models were similar to that expected from the simulations.

Model	Nb of parameters	OFV	dOFV vs Ref
M_0	10	-369.2	+27.9
M_{CRCL}	11	-397.1	Ref
M_{WT}	11	-392.1	+5

Cross-validation

To estimate the predictive performances of the three models (M_0, M_{CRCL}, M_{WT}) a ten-fold cross-validation was performed (Ribbing et al. 2007). Ten training data sets were constructed by pooling nine of the ten parts, and the predictive performances assessed on the remaining part, the validation data set. The OFV of the ten validation datasets were averaged to obtain the cross-validation OFV.

Model	Mean validation OFV
M_0	-293.4
M_{CRCL}	-321.6
M_{WT}	-316.2

Discussion

The simulations indicated that the power to discriminate against no covariate was high, ca 98%, and it was still high but slightly lower for discrimination between the two covariates, ca 93%. In this situation, the prior knowledge of the renal route being important is allowed to weight in on the otherwise data driven selection. The prior value of 0.75 allow us to specify a preference, but not complete reliance, of CRCL as the better model. In this case, the simulations performed provided a good assessment of the discriminatory power of the data with respect to the covariate association with clearance. This is evidenced by similar differences in OFV in the simulations as in the real data set. The cross-validation results indicate that the CRCL model has the best predictive performance with respect to OFV.

References

Karlsson, Mats O, E Niclas Jonsson, Curtis G Wiltse, and Janet R Wade. 1998. "Assumption Testing in Population Pharmacokinetic Models: Illustrated with an Analysis of Moxonidine Data from Congestive Heart Failure Patients." *Journal of Pharmacokinetics and Biopharmaceutics* 26 (2). Springer: 207–46.

Ribbing, Jakob, Joakim Nyberg, Ola Caster, and E Niclas Jonsson. 2007. "The Lasso—a Novel Method for Predictive Covariate Model Building in Nonlinear Mixed Effects Models." *Journal of Pharmacokinetics and Pharmacodynamics* 34 (4). Springer: 485–517.
